# Full-Digital Workflow for TMDs Management: A Case Series

**DOI:** 10.3390/healthcare11060790

**Published:** 2023-03-08

**Authors:** Simona Tecco, Alessandro Nota, Laura Pittari, Chiara Clerici, Francesco Mangano, Enrico Felice Gherlone

**Affiliations:** 1 Dental School, Vita-Salute San Raffaele University, I.R.C.C.S. San Raffaele Hospital, Via Olgettina 48, 20132 Milan, Italy; 2 Department of Prevention and Communal Dentistry, Sechenov First Moscow State Medical University, 119435 Moscow, Russia

**Keywords:** TMD, digital dentistry, gnathology, splint, ModJaw, kinematics, bite registration, occlusion, rest vertical dimension, case series

## Abstract

Temporomandibular joint disorders (TMDs) have always been the subject of studies due to the difficult management of symptoms and the complex stabilization of the so-called therapeutic position. In this effort, digital technologies open new opportunities for such planning, allowing the clinician to digitally assess the situation and verify the stability of the new position from a functional point of view. The present case series shows examples of preliminary full-digital planning of treatment in TMDs patients made with the preliminary evaluation of the kinematic activity of the mandible through a digital device (Tech in motion™, ModJaw, Villeurbanne, France). Three TMD clinical cases are analyzed with full-digital techniques and workflow. A personalized treatment for each case was digitally planned on the base of proper kinematic tracings recorded for each patient, and intraoral appliances were digitally customized through a full-digital or semi-digital workflow. The digitalization of mandibular kinematic gave us the possibility of making a more “aware” diagnosis, especially in a dynamic key, and then it allowed a faster realization and execution of the intraoral appliance through a digital workflow, memorizing the therapeutic position and early checking the device, before its realization, on the real kinematics of the patient.

## 1. Introduction

In complex clinical cases that request gnathological, orthodontic, restorative, and prosthetic rehabilitation, preliminary simultaneous planning of all stages of treatment immediately from the initial situation to the result is mandatory to minimize the probability of errors, increasing the predictability of the entire result while also improving the interdisciplinary work of the involved specialists [1]. Complex dental rehabilitation often begins with a preliminary use of an occlusal appliance designed to enable the patient to have a different mandibular position, which will then eventually be used for definitive rehabilitation.

The first step of the entire rehabilitation is to establish the mandibular position and occlusal vertical dimension, which is strategic data to plan all the next steps [1,2]. In this effort, digital technologies open new opportunities for such planning, allowing the clinician to digitally assess the initial clinical condition and verify the stability of the planned mandibular repositioning from a kinematic point of view. Mandibular repositioning is studied so that it can guarantee stable mandibular contact in the maximum intercuspation and a symmetrical path of the mandible in the laterality and in the protrusion. 

Verifying the kinematics of the new mandibular position is one of the most complex clinical problems. Clinical gnathology has always aimed to offer “personalized” solutions to the patient, aiming to identify for each patient a new mandibular position that is more stable from a neuro-muscular point of view, to be achieved by an oral appliance through a guided or spontaneous mandibular repositioning. 

Mandible repositioning has often been hypothesized as a forward–downward reposition. This is because it has been shown that condyles with osteophyte formations (therefore with chronic joint pathology) are, in general, increasingly displaced postero-superiorly in the glenoid fossa when they are in a position of usual occlusion compared with the condyles of subjects without bone alteration [3]. This suggested that the uncorrected condylar position could influence its shape with morphological alteration [3]. Condylar displacement can also be observed in adolescents without bone alteration [4]. Consequently, it was reasonably supposed that one of the causes of intra-articular pain—not only in disc displacement patients but also in chronic and/or parafunctional arthrosis—might be due to a compression of the bilaminar zone caused by the uncorrected position of condyles [5]. One of the radiological manifestations of this condition is the decrease in the superior and posterior amplitude of the intra-articular space. Therefore, from a therapeutic point of view, it was supposed that if the intra-articular space is increased, allowing for a more fluid condylar translation beyond the disc surface, then irregularities and position anomalies are supposed to disappear [5].

Conventional standardized methods to achieve mandibular repositioning are clinical tests and/or the visualization of the joint anatomy in MRI, which is used to identify the correct position of the condyle in the fossa [6,7,8]. However, in a recent systematic review [9], the authors pointed out that most of these available techniques are useful for routine clinical use. Nevertheless, they are empirical in nature, often controversial, and lacking scientific support. Hence, the main conclusion of that review was that there is no single accurate method to establish mandibular repositioning.

**Figure 1:**
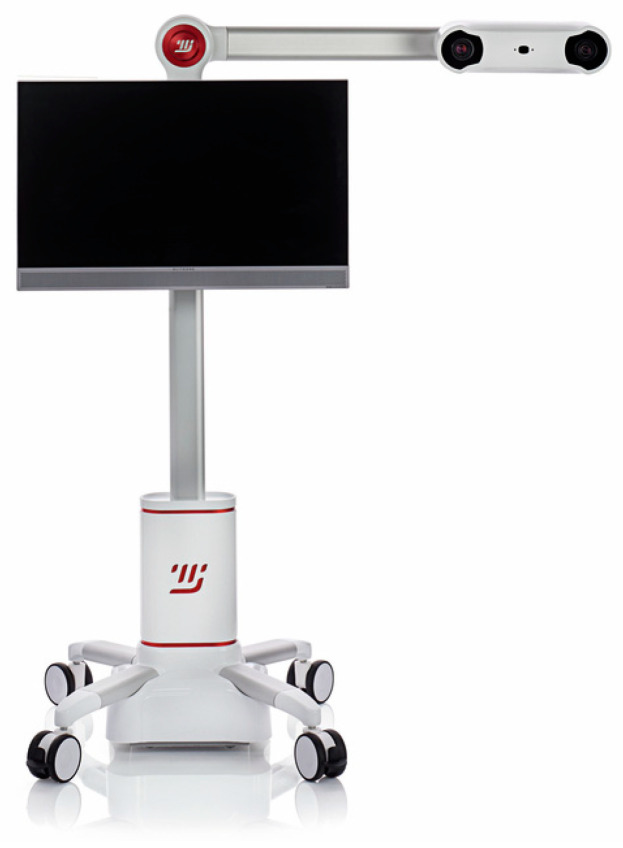
The ModJaw device with a camera based on 3D stereophotogrammetry to record mandibular movements on STL models.

Today, digital technology can come to help clinicians for this purpose, as it allows previsualization of the condylar position and its movements—both in the habitual intercuspation and after its repositioning—allowing a more “rational” choice of a stable mandibular position to plan the entire treatment. This is possible through the preliminary evaluation of the kinematic activity of the mandible by using a recently developed technological device (Tech in motion™, ModJaw, Villeurbanne, France) that uses a digital camera and reproduces real mandibular movements on the. STL files of the arches. These are useful to evaluate condylar tracings, improve the accuracy of the diagnosis, and make the design of an oral appliance ideally customized from the functional aspect. This device is available thanks to the development of two sectors of technological research: that linked to the recording of movements using a sophisticated camera based on 3D stereophotogrammetry (Figure 1), and that concerning visualization of digital models of the dental arches, which can also be matched with the Cone Beam Computed Tomography (CBCT) of the patient (in the most recent version 4.0 of ModJaw), integrating the kinematic evaluation with the visualization of the temporomandibular joints (TMJ) and increasing the precision and predictability of the result in complex cases.

In the present case series, three clinical cases are analyzed with full-digital techniques and workflow. Each patient was asked to sign an informed consent form, and the “parere09/int/2023” gnathological protocol approved by the IRCCS San Raffaele Hospital (Milan, Italy) Ethics Committee was followed by personalized treatment for each patient. The case was digitally planned on the basis of proper kinematic tracings recorded for each patient, and oral appliances were digitally customized through a full-digital or semi-digital workflow. 

## 2. Case Reports

### 2.1. Case 1

**Figure 2:**
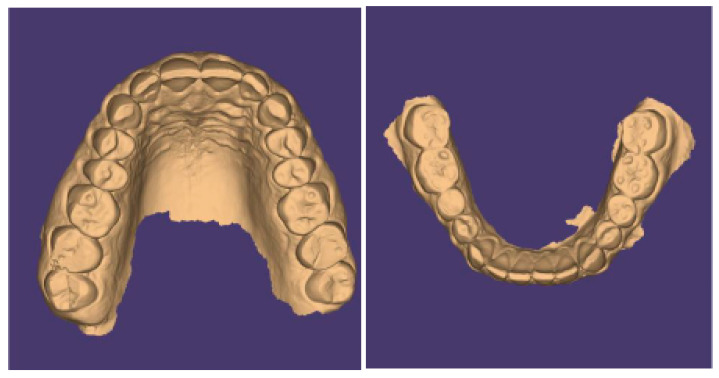
Occlusal view (STL files).

The present case is from a 45-year-old woman affected by TMDs, based on DC/TMD [10]. In her medical history, the patient reported that she was aware of suffering for years from awake bruxism, which manifested itself above all during moments of concentration and intellectual work, associated with headache in the area of the temples, with pain in the masseter muscles and, occasionally, with bilateral swelling of the temple areas. The diagnosis was, therefore, myofascial pain of the masseter muscles with associated headache, right and left disc displacement without reduction, and a slightly reduced opening path [11,12]. Intraoral clinical examination evidenced alteration of the occlusal plane, with numerous wear facets and completely abraded posterior teeth with uncovering of the dentin (Figure 2).

**Figure 3:**
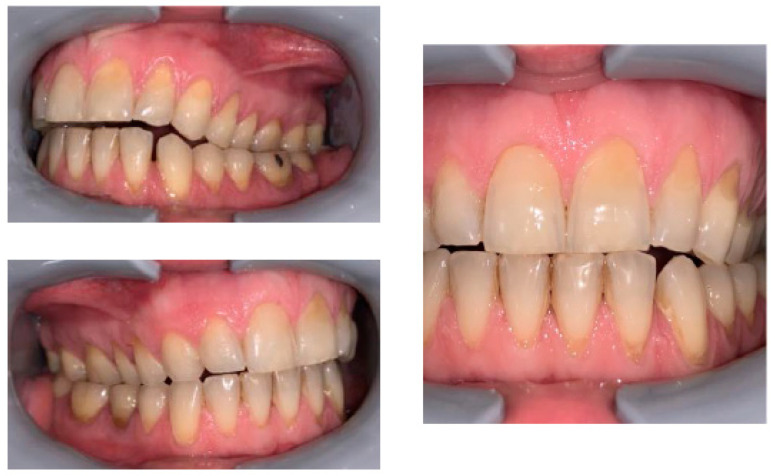
Occlusal relationships.

The occlusal relationships are shown in Figure 3.

**Figure 4:**
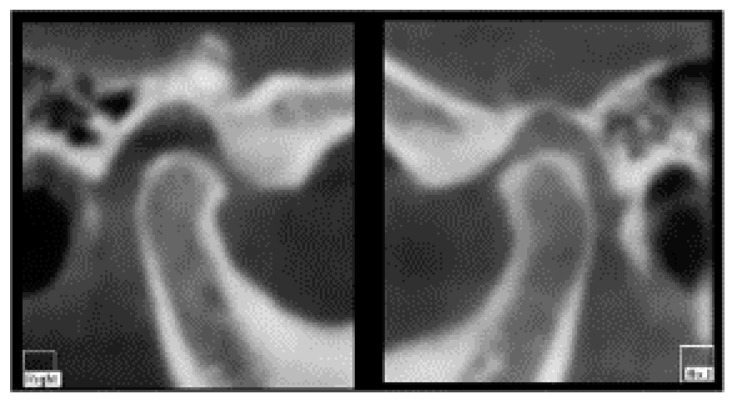
CBCT view of temporomandibular joints: no serious signs of morphological condylar involvement and a slight reduction of the intra-articular space.

CBCT does not show serious signs of morphological condylar involvement resulting from chronic inflammation [13], and a slight reduction of the intra-articular space was observed (Figure 4).

**Figure 5:**
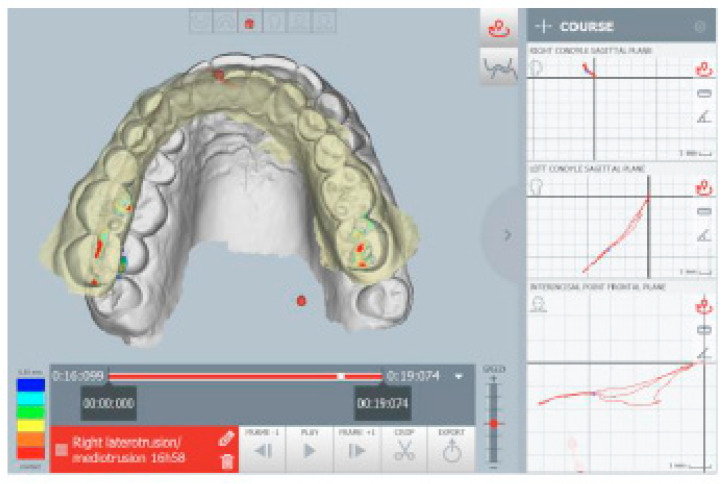
Right lateral movement.

**Figure 6:**
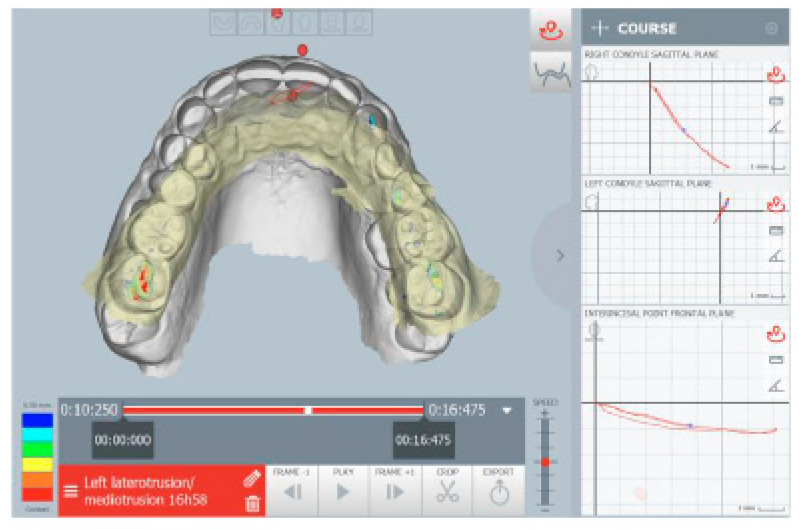
Left lateral movement.

**Figure 7:**
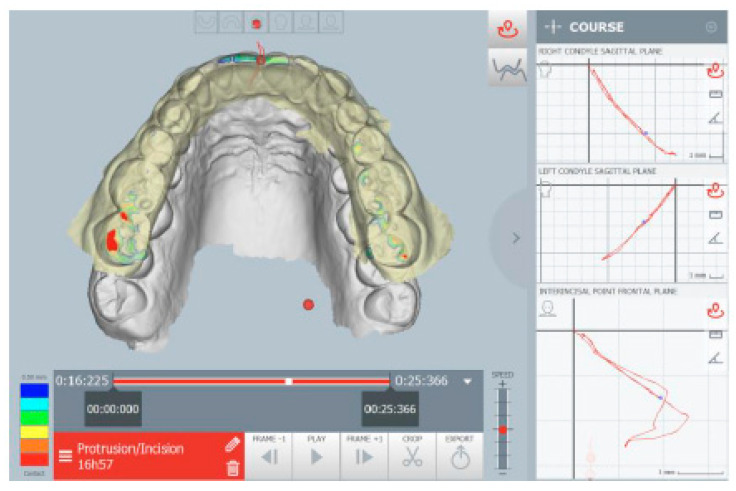
Occlusal contacts during mandibular protrusion movement. The red points indicate an abnormal contact during protrusion.

**Figure 8:**
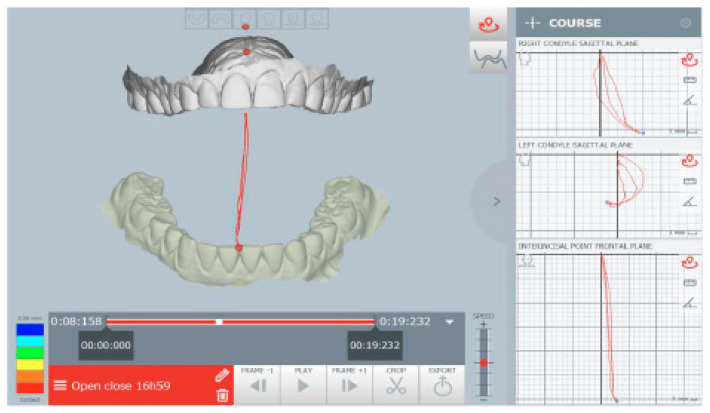
Opening tracing.

The kinematic examination of the mandibular movements was performed using the ModJaw device, as shown in Figure 5, Figure 6, Figure 7 and Figure 8.

Figure 5 highlights the right lateral movement, during which the total absence of the canine guides is noted, with contacts present only at the level of the molars. All wrong contacts were identified in real time during the first visit. 

Figure 6 shows left lateral movement, with the absence of a canine guide and a completely flat path.

The kinematic examination [14] of the protrusion movement also showed the total flattening of the incisor guide with interocclusal contacts present on the molars, especially on the right, during the protrusion path (Figure 7).

Figure 8 shows a deflection of the opening tracing at the maximum opening to the left, with a reduction in the opening amplitude (about 30 mm).

**Figure 9:**
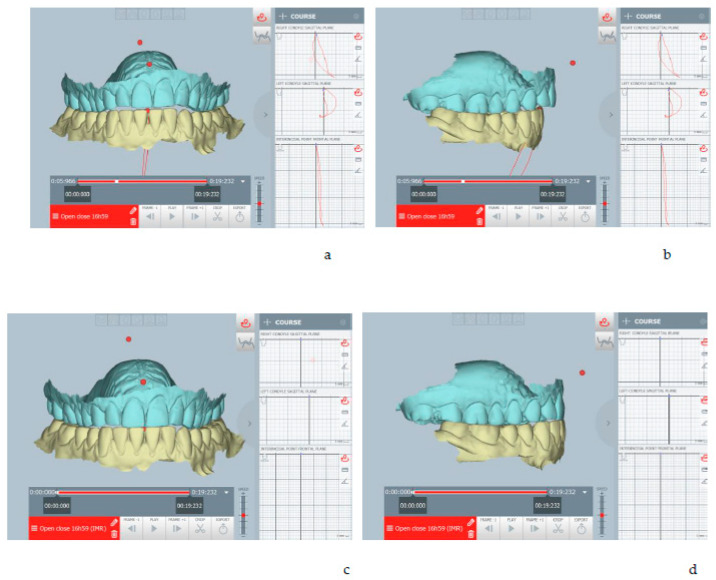
(**a**,**b**) The habitual occlusion position, and (**c**,**d**) the therapeutic position obtained after digital planning, repositioning the condyles forward and downward, and re-centering the midline. The therapeutic position was planned in real time during the patient’s diagnostic checkup (one-day full-digital workflow).

The mandibular repositioning after digital planning brought condyles forward and downward by about 1 mm and re-entering the midline. The STL file of the arches in the therapeutic position highlights the gap between the dental arches (Figure 9).

**Figure 10:**
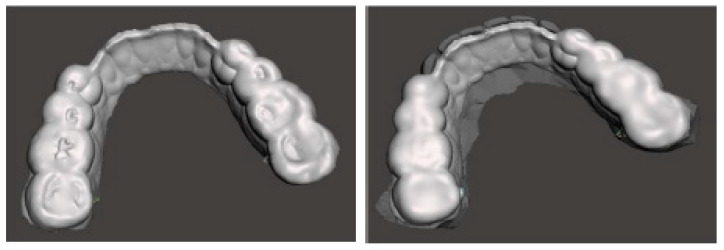
Lower arch device (M.O.R.A. appliance) to be used during the day thanks to its comfortability and lower impact on social life.

**Figure 11:**
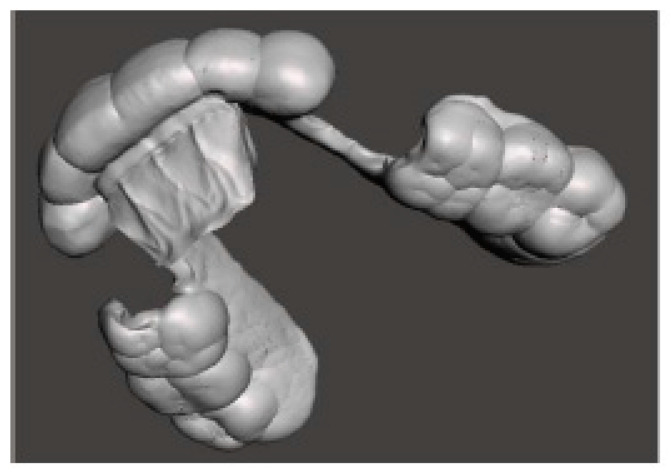
Upper arch device to be used during the night with a design that allows it to maintain a therapeutic position during sleep.

The therapeutic position was therefore recorded and exported on an STL file of the dental arches (Figure 9), which was digitally sent to the laboratory (Dentallaboratory, Brescia, Italy) on the same day. In this case, a device for the lower arch and one for the upper arch was designed, represented in Figure 10 and Figure 11. The upper device is represented in Figure 11.

**Figure 12:**
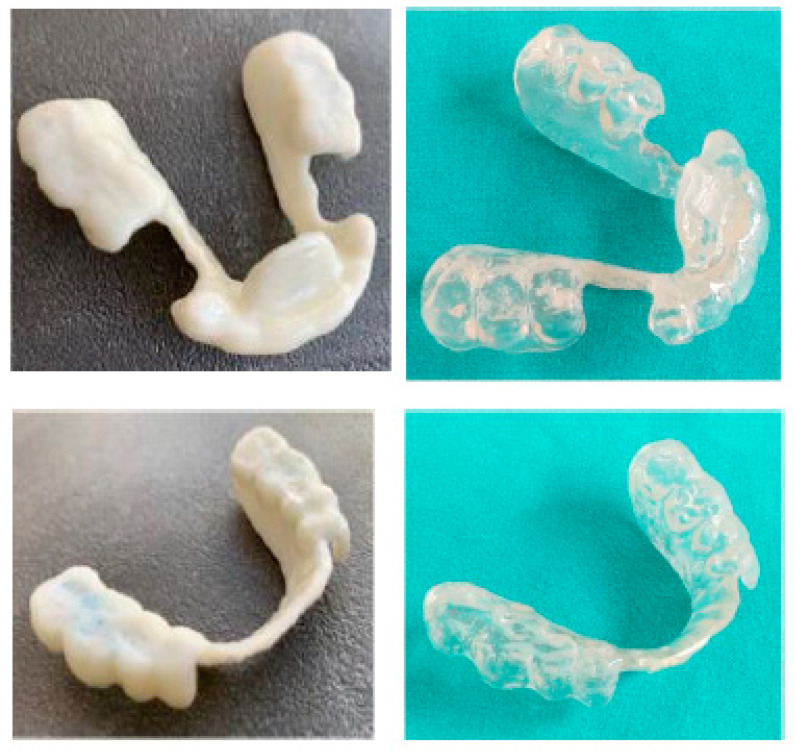
White or transparent material.

Meshmixer (Autodesk Inc., San Rafael, CA, USA) software was used in the laboratory for the design and Simplify3D (Simplify3D, Cincinnati, OH, USA) for the printing of the devices. During the design, full attention was paid to the phase of checking the minimum thicknesses and retentive extensions of the appliances with Rhinoceros software (Robert McNeel and Associates, Seattle, WA, USA). The printing phase took place with a 3D printer (Prometeo2, 3D Makerlab, Roncadelle (Brescia), Italy) with optimal intensity, and the devices were then subjected to washing in IPA with special products. Subsequently, the supports were removed. The material used for printing is biocompatible, certified as a medical material, and white or transparent (ABS MED, 3D Makerlab, Roncadelle (Brescia), Italy) (Figure 12).

**Figure 13:**
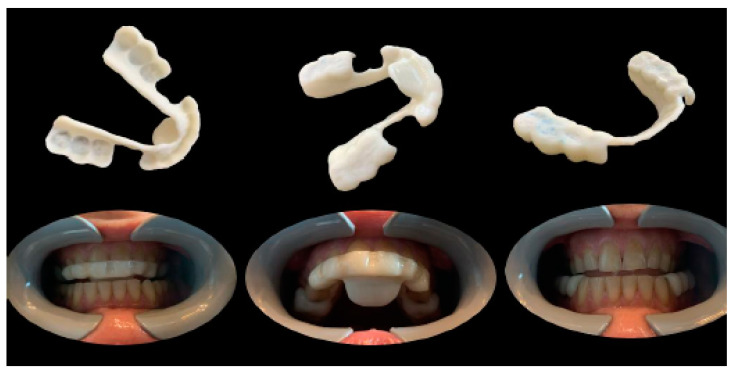
Digitally printed devices, without metal parts, made for the lower and upper arches on the basis of the therapeutic position identified during the diagnostic checkup. Ideally, the devices can be designed and printed on the same day of the visit (one-day full-digital workflow).

In this case, the patient received the devices a few days after her first visit, as all the information necessary for printing had been sent to the laboratory on the day of the visit. Ideally, this full-digital workflow allows a gnathological bite to be delivered to the patient on the day of the visit, which could further reduce the work time. Figure 13 shows the devices worn by the patient.

**Figure 14:**
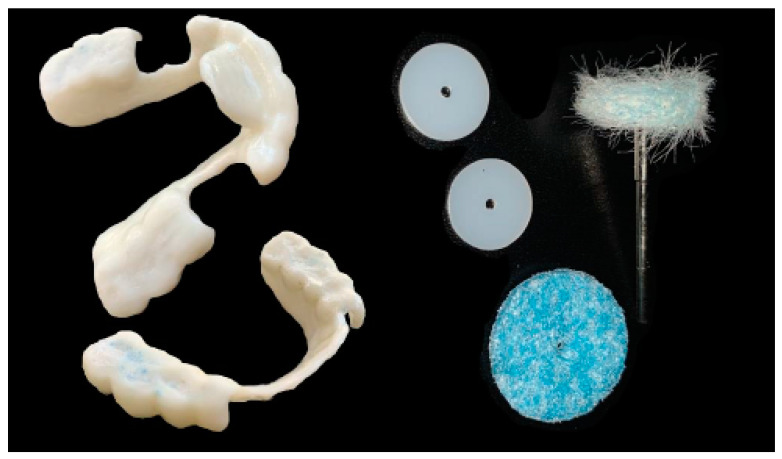
For any adjustments, only finishing tips should be used.

Digitally designed devices are comfortable and precise and require minimal chairside adjustments that can only be performed with trimming tips (Figure 14).

In this clinical case, the patient was asked to wear the upper arch device at night and the lower arch device during the day, except during meals, for a period of at least 4 months, after which, thanks to the improvement of the symptoms, the reduction of the use of the lower arch device was recommended, slowly and progressively, until the device is worn during the day only when needed. Currently, the patient generally wears the device only at night and continues with the monitoring of the parafunction [15,16,17]. The present clinical case was managed through a full-digital workflow from the diagnosis to the printing of the devices. 

### 2.2. Case 2

**Figure 15:**
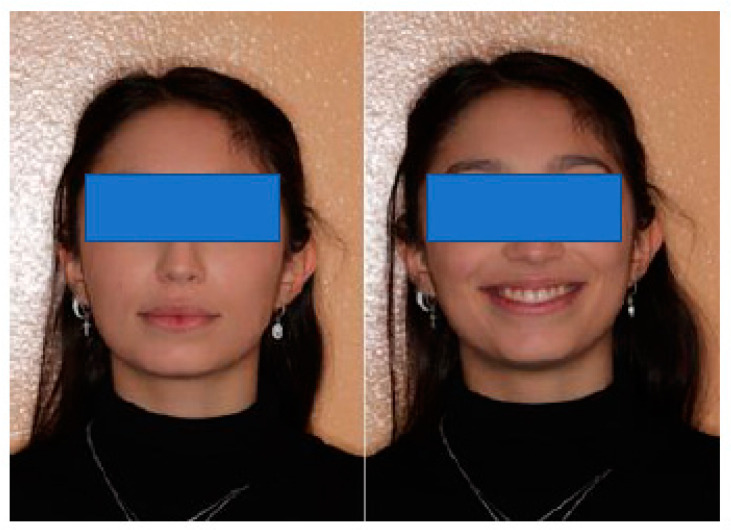
Extra-oral facial views.

A 22-year-old woman presented with discomfort during chewing, joint click, and headache that began following a facial trauma suffered 2 years before (at the right TMJ) during a dance session. Of note was the fact that she is a carrier of Mediterranean anemia. In the past, she has undergone orthodontic treatment and revealed open-locking episodes. On extraoral clinical examination, she has a slight deviation of the chin to the right (Figure 15). 

**Figure 16:**
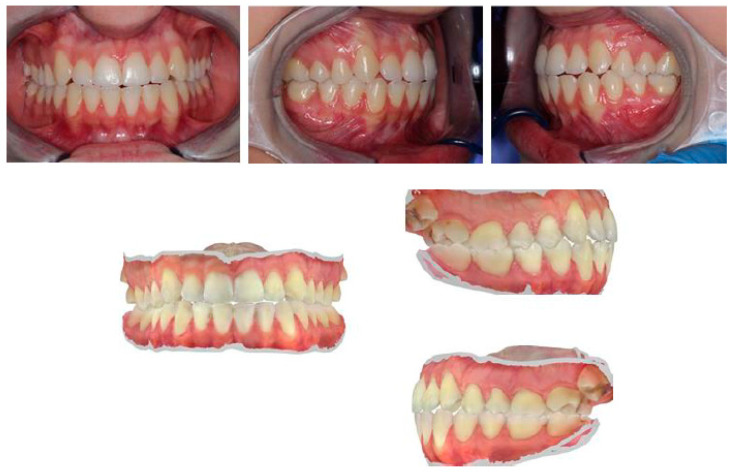
Intraoral photos and intraoral scans show occlusal relationships: class I molar on the right and left and lower midline deviated to the right compared with the upper.

Intraoral occlusal relationships are shown in Figure 16.

**Figure 17:**
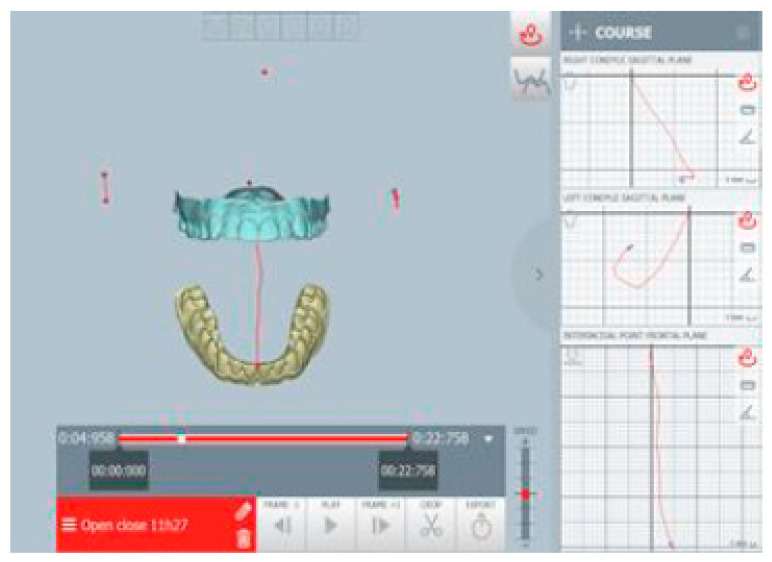
Mandibular kinematic during opening. There was a normal opening amplitude, with deflection to the left side. There were some slight deviations to the right and left, not always present during the opening movements. The right condylar path showed hypermobility in maximum opening.

On clinical examination, she revealed joint clicks on the right TMJ and also on the left TMJ and pain on palpation of the masticatory muscles, especially on the right. Kinematic analysis of mandibular movements revealed functional problems. There is a normal opening amplitude, with deflection to the left side (Figure 17). 

**Figure 18:**
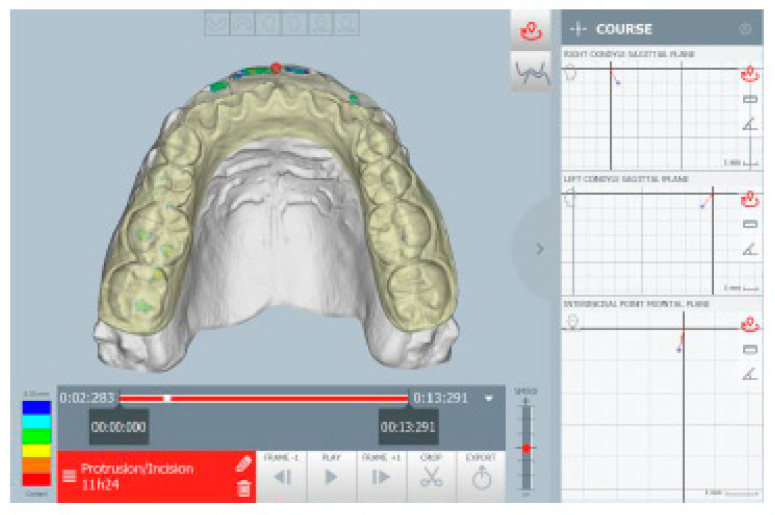
The protrusion tracing.

**Figure 19:**
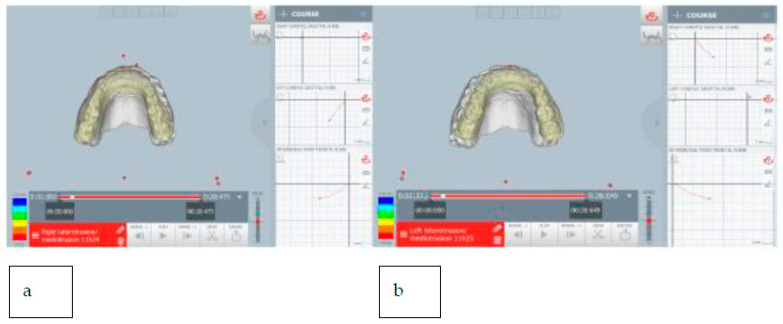
Lateral tracings, right (**a**) and left (**b**) sides.

There were some slight deviations to the right and left, not always present during the opening movements. The right condylar path showed hypermobility in the maximum opening (Figure 17). The protrusion tracing showed the absence of incisal guidance (head-to-head incisors) (Figure 18). In addition, the right lateral guide was performed by the group of teeth 16 to 14, with pre-contact between teeth 26 and 36. The left lateral guide was performed by the group from teeth 26 to 34 with pre-contact between teeth 16 and 46. (Figure 19).

**Figure 20:**
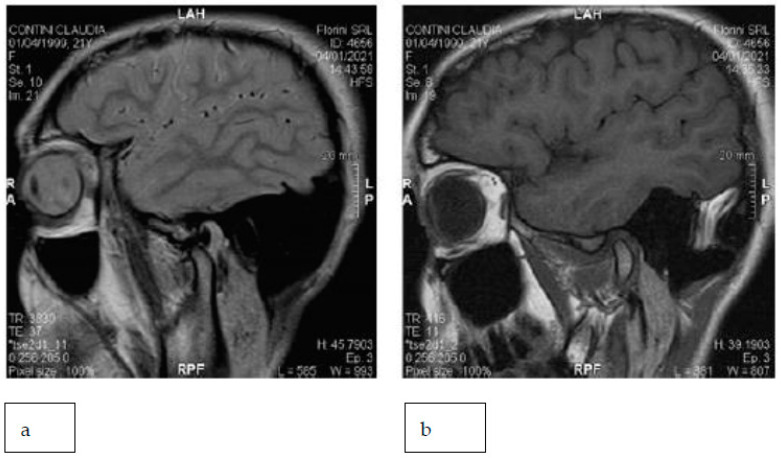
The right TMJ with an open (**a**) and closed (**b**) mouth.

MRI of the TMJs with closed and open mouths showed disc displacement with a reduction in both TMJs (Figure 20).

**Figure 21:**
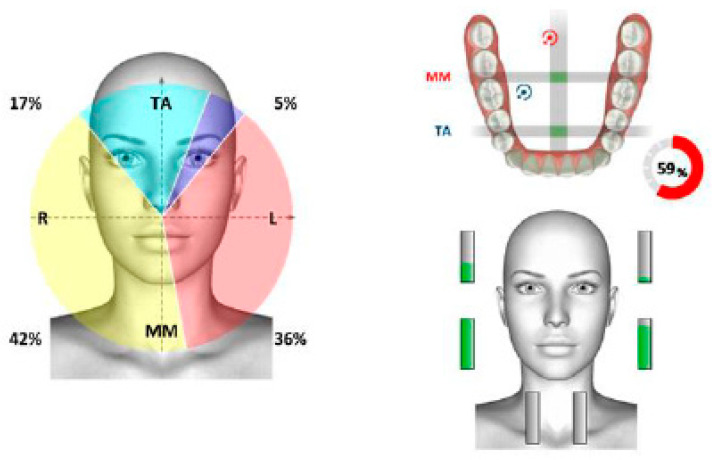
Surface electromyography in mandibular rest position.

Surface electromyography of the masticatory muscles confirmed asymmetry, with a prevalence of muscular work of the anterior temporal muscles on the right (Figure 21).

**Figure 22:**
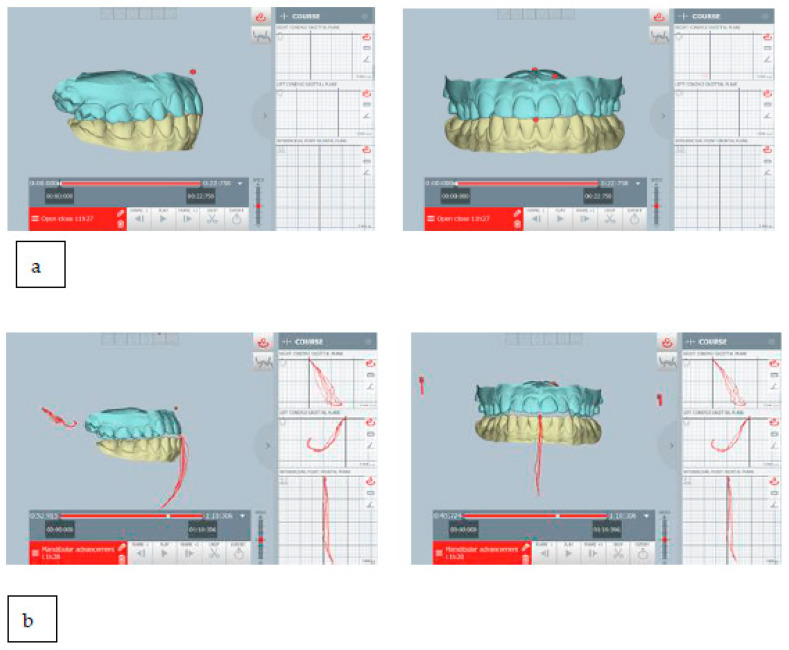
(**a**) The habitual occlusion position; (**b**) mandibular repositioning for intraoral appliance; ModJaw allowed to evaluate of kinematic tracings of the condyle from the therapeutic position. The mandible was repositioned forward about 2 mm and downward about 1 mm.

The mandibular repositioning was digitally planned after evaluating its movements through the ModJaw device. In the present case, the mandible was repositioned forward about 2 mm and downward about 1 mm (Figure 22).

**Figure 23:**
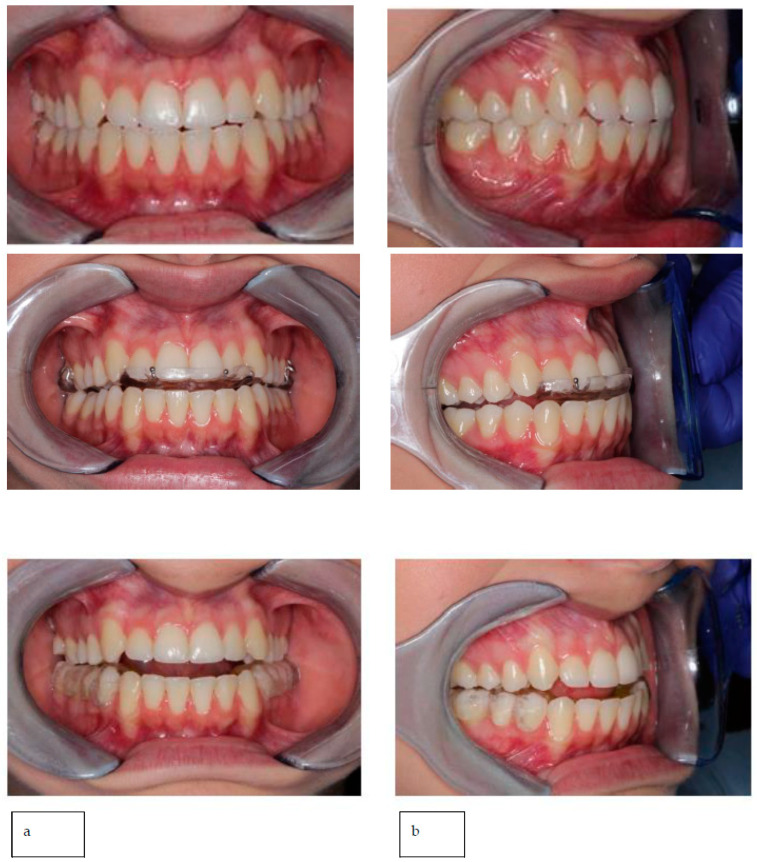
(**a**) Habitual occlusion; (**b**) upper device to be used during the night; and (**c**) lower device to be used during the day.

The position was planned to achieve an increase of intra-articular space and maintain a straight open tracing. Therefore, the position was recorded, and all the acquired data were digitally shared with the dental laboratory. In the present case, the lower occlusal device was digitally designed and printed (with the same procedure as the previous case reported), while the upper device was produced using a traditional method (Figure 23).

In the present case, the treatment with occlusal appliances consisted of repositioning appliances in the upper and lower arches, to be alternatively worn night and day for about 6 months. In addition, mandibular stabilization exercises were also recommended for the management of joint hypermobility. Finally, digital monitoring of the daytime parafunction with Brux-app (WMA, Lucca, Italy) [18] was recommended in order to manage a progressive reduction of the use of the daytime device. 

### 2.3. Case 3

**Figure 24:**
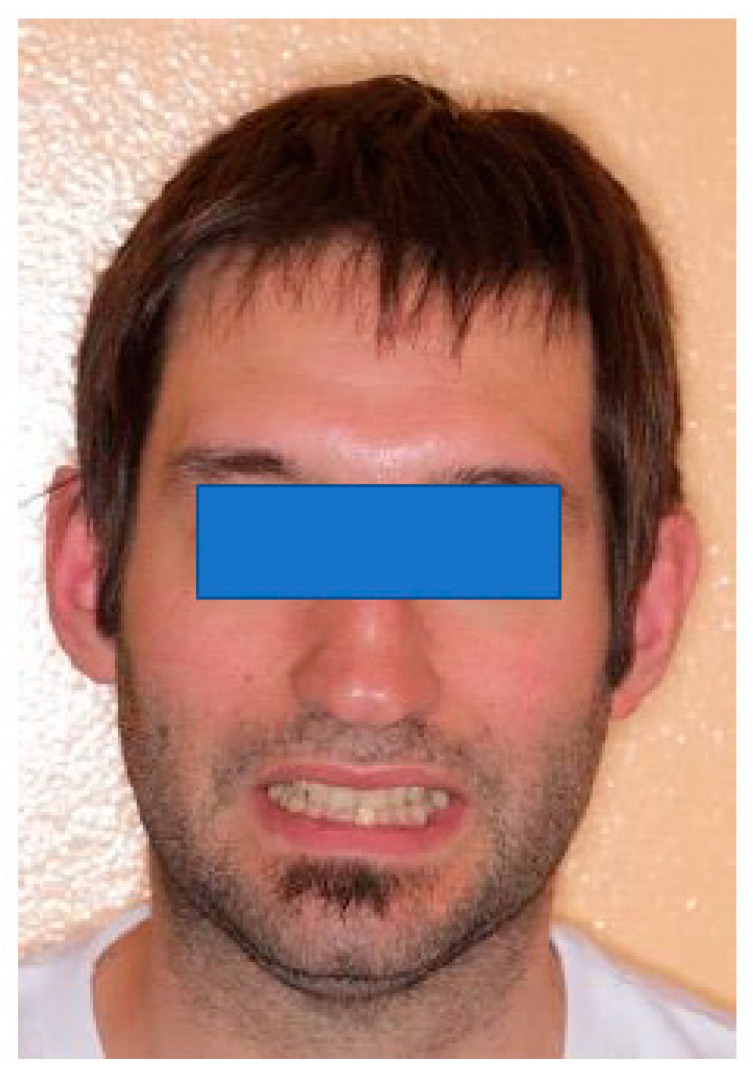
Extraoral examination reveals upper and lower midlines deviated 1 mm to the right from the midline of the face, with a disharmonious smile.

A 35-year-old male came to our attention due to pain in the posterior dental sectors and TMJ area, which started about 6 months prior. The pain was initially episodic and then became continuous. Medical history revealed that the patient was suffering from headaches, episodes of dizziness, and psychological complaints due to anxiety. The patient reported habitual grinding. The extraoral view is shown in Figure 24.

**Figure 25:**
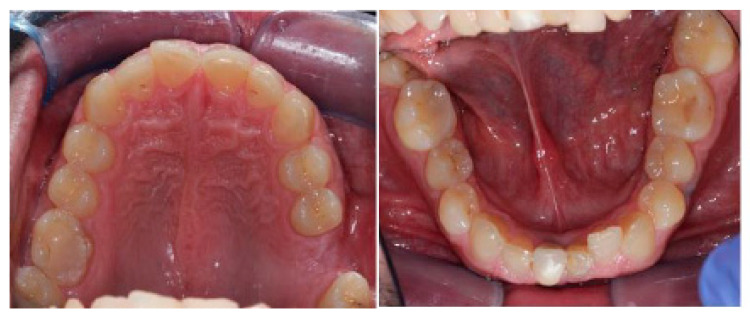

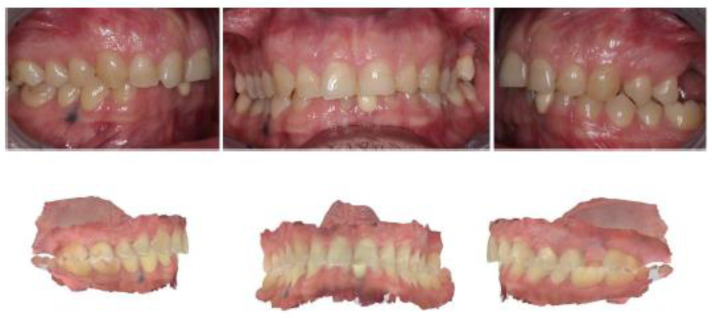
Intraoral views show increased overbite (4–5 mm), overjet of about 1 mm, class I molar on the right, class II canine on the right, and class I canine on the left. There is evidence of widespread wear facets and a mild gingival recession on tooth 33.

**Figure 26:**
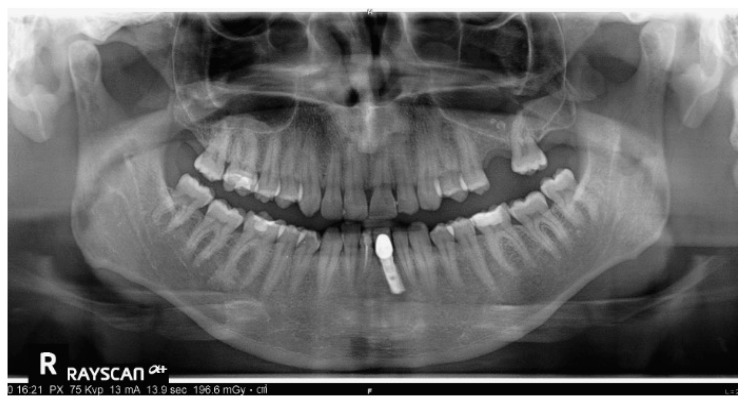
Absence of element 26, implant-prosthesis of element 31, and condylar asymmetry. It was indicated to investigate the diagnostic with an X-ray of elements 36 and 46.

Intraoral views are shown in Figure 25, and Figure 26 reports orthopantomography: the absence of element 26, implant-prosthesis of element 31, and condylar asymmetry. It was indicated to investigate the diagnostic with an X-ray of elements 3.6 and 4.6.

The patient revealed pain on palpation of the right and left TMJs. Palpation of the masticatory muscles revealed pain in the lateral pterygoid muscle area, both right and left, and generalized discomfort of the masseter and suprahyoid muscles.

**Figure 27:**
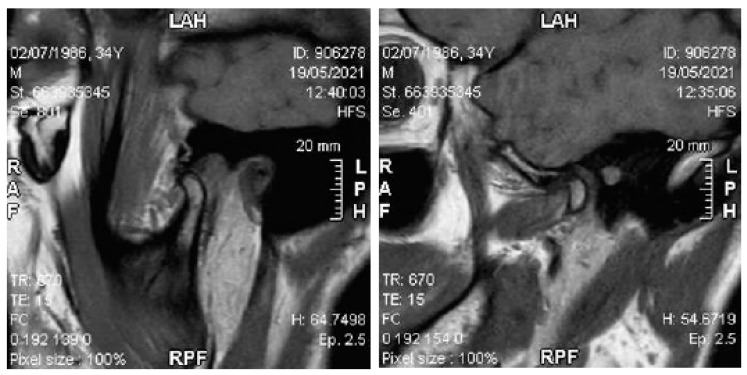
MRI images with a closed and open mouth.

The patient showed a small opening width, approximately 25–30 mm, without pain. Lateral movements were within the norm. MRI images of the TMJ show anterior displacement of the disc with reduction (Figure 27).

**Figure 28:**
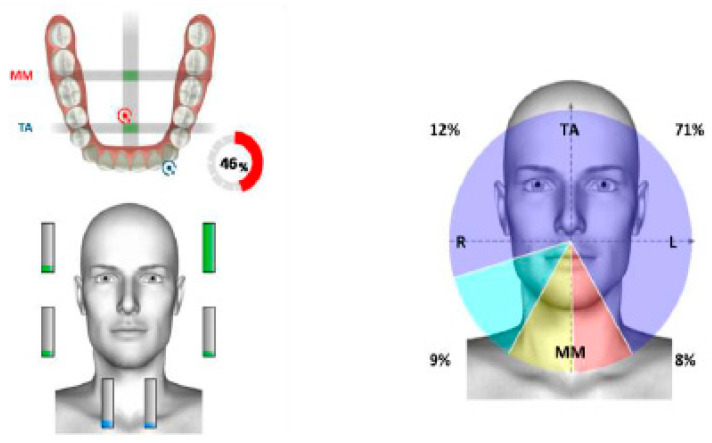
Surface electromyography.

The functional evaluation of the masticatory muscles evidenced the prevalence of the anterior temporal on the left, with functional torsion (Figure 28).

**Figure 29:**
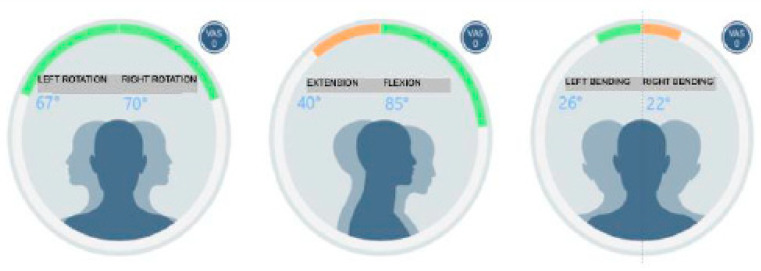
Evaluation of cervical movements.

In the present case, the evaluation of cervical movements (BAIOBIT—Rivelo SRL, BTS Bioengineering group) showed a reduction in the amplitude of the extension movements of the head and bending on the right side (inclination) [19] (Figure 29).

**Figure 30:**
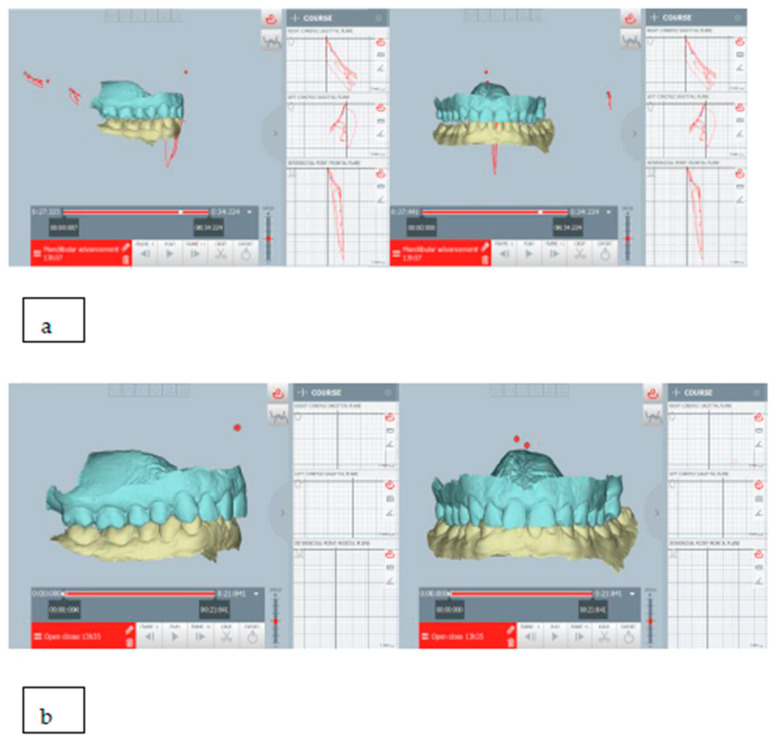
(**a**) The planned therapeutic position: ModJaw allowed the evaluation of kinematic tracings of the condyle from the therapeutic position and (**b**) the habitual occlusion position; as seen, it was planned as sliding downward and forward repositioning of the mandible.

For this patient, oral devices were indicated for the management of chronic parafunction (it was recommended they use a night-splint and, if necessary, a day-splint). The therapeutic position was digitally planned with a sliding condyle repositioning downward and forward, and it is shown in Figure 30. 

Therapy for the management of anxiety disorders and a cycle of physiotherapy in the cervical area was also recommended.

## 3. Discussion

In these cases, the first step of the entire rehabilitation after a proper diagnosis was to establish a mandibular therapeutic position that is fundamental to planning all the next ones. To this aim, today, digital technologies offer “personalized” solutions to identify, for each patient, a stable mandibular position that is digitally planned on the basis of functional tracings recorded, which is achieved through guided or spontaneous mandibular repositioning through intraoral appliances digitally customized. Previous studies tried to compare the digital methods of construction of a splint with the conventional ones without detecting significant differences between the two methods [20,21]. 

In a recent study comparing three-dimensional printing to subtractive computer-aided manufacturing, the first offered a promising alternative to CAM in terms of production accuracy and therapeutic success at reduced costs [22]. In that study, the substrates were subjected to mastication simulation (120,000 cycles, 37 °C, 50 N, 1.3 Hz) as opposed to enamel antagonists. Then, the wear was measured through matching of the scanned substrates before and after aging, and damage patterns were categorized and evaluated based on microscopic examinations.

There are several different methods described in the literature to obtain a mandibular centric relation, including leaf gauge, tongue tip to the soft palate, gothic arch tracing, myo-monitor, Roth power bite, chin-point guidance, bimanual manipulation, and long-term deprogrammer with passive muscle contraction [23]. 

Conventional procedures for determining mandibular repositioning also consisted of the visualization of the joint anatomy in MRI images, used to identify a correct position of the condyle in the fossa, associated with a stable occlusal relationship in static [6,7,8].

In a recent systematic review [9], the authors pointed out that most of these available conventional procedures are useful for routine clinical use. However, they are empirical in nature, often controversial, and lacking scientific support. Hence, the main conclusion of that review was that there is no single accurate method for determining the vertical occlusal dimension.

In the present clinical cases, no radiographic monitoring of the condylar position was performed for the patients, different from clinical cases reported in the literature [24] in which a second CBCT was taken. In a recent study performed with CBCTs taken before and after treatment, it was observed that the changes in the dimension of anterior, superior, posterior, and medial joint spaces after the end of the treatment with a splint and physiotherapy in patients with TMD are often null [25,26]. Occlusal splint therapy with physiotherapy did not significantly change the dimension of joint spaces nor place the mandibular condyles into the centric relation. Another study performed on MRI confirmed that the use of a repositioning splint resulted in forward and downward condyle movement, resulting in an ideal spatial disc-condyle relationship, but the stability of this relationship could not be maintained in the majority of TMJs if the splint is removed [27]. 

Findings explain the good short-term clinical outcomes with anterior repositioning splints and their relatively lower efficacy in the long term. Thus, treatment of patients with TMD should not aim at the gnathological concept of placing the mandibular condyles into centric relation because centric relation appears not to be mandatory to achieve successful clinical results of treatment in patients with TMD. According to this, the present clinical cases were clinically followed during their follow-up, no matter the condyle’s repositioning obtained. In the present case reports, all three patients achieved a significant improvement in their symptoms in about 6–8 months. They wore their night-splint over time but reduced the use of their day-splint after 3–4 months after the beginning of treatment, adopting its usage only “when needed”, after which they were followed for cognitive behavior therapy. 

The present work has some limits; first of all, the results obtained are still presented on a case-series basis, while the clinical efficacy of occlusal splints still seems to be controversial in the scientific literature. Although some of the authors emphasize the positive effects of the occlusal splints on TMD pain reduction, as well as on increasing maximum mouth opening [28], according to other authors, the efficacy of occlusal splints is of either low or very low quality and further studies are needed [29]. Another limit is represented by the lack of follow-up for the 2nd and 3rd cases. Furthermore, the present cases do not show the integration of the CBCT with the digital analysis, as it is reserved for the most complex cases according to the ALARA principle. 

## 4. Conclusions

The digital workflow has many advantages:

- The possibility of making a more “aware” diagnosis, especially in a dynamic key, on the most complex cases;
- Faster realization and execution of the intraoral appliance;
- The possibility of digitally memorizing the therapeutic position and virtually checking the device before its production on the real kinematics of the patient, without the need for chairside adjustments of the device;
- The exchangeability of the planned mandibular position and its kinematics with other colleagues within the interdisciplinary dental team who will have to deal with the prosthetic and/or conservative part of finalization;
- The 3D printing/milling allows the production of a duplicate of the device if needed and improves comfort and related acceptance thanks to the rounded edges and beveled edges.

## Data Availability

Archived datasets analyzed and generated during this study are available via email after requesting them from the authors (via email).
